# Phylogenetic Diversity and Physiological Roles of Plant Monovalent Cation/H^+^ Antiporters

**DOI:** 10.3389/fpls.2020.573564

**Published:** 2020-10-06

**Authors:** Stanislav V. Isayenkov, Siarhei A. Dabravolski, Ting Pan, Sergey Shabala

**Affiliations:** ^1^ International Research Centre for Environmental Membrane Biology, Foshan University, Foshan, China; ^2^ Department of Plant Food Products and Biofortification, Institute of Food Biotechnology and Genomics NAS of Ukraine, Kyiv, Ukraine; ^3^ Department of Clinical Diagnostics, Vitebsk State Academy of Veterinary Medicine [UO VGAVM], Vitebsk, Belarus; ^4^ Tasmanian Institute of Agriculture, University of Tasmania, Hobart, TAS, Australia

**Keywords:** NhaP, Na^+^/H^+^ exchanger, NHE, K^+^-efflux antiporter, KEA, cation/H^+^ exchanger, CHX, molecular phylogeny

## Abstract

The processes of plant nutrition, stress tolerance, plant growth, and development are strongly dependent on transport of mineral nutrients across cellular membranes. Plant membrane transporters are key components of these processes. Among various membrane transport proteins, the monovalent cation proton antiporter (CPA) superfamily mediates a broad range of physiological and developmental processes such as ion and pH homeostasis, development of reproductive organs, chloroplast operation, and plant adaptation to drought and salt stresses. CPA family includes plasma membrane-bound Na^+^/H^+^ exchanger (NhaP) and intracellular Na^+^/H^+^ exchanger NHE (NHX), K^+^ efflux antiporter (KEA), and cation/H^+^ exchanger (CHX) family proteins. In this review, we have completed the phylogenetic inventory of CPA transporters and undertaken a comprehensive evolutionary analysis of their development. Compared with previous studies, we have significantly extended the range of plant species, including green and red algae and Acrogymnospermae into phylogenetic analysis. Our data suggest that the multiplication and complexation of CPA isoforms during evolution is related to land colonisation by higher plants and associated with an increase of different tissue types and development of reproductive organs. The new data extended the number of clades for all groups of CPAs, including those for NhaP/SOS, NHE/NHX, KEA, and CHX. We also critically evaluate the latest findings on the biological role, physiological functions and regulation of CPA transporters in relation to their structure and phylogenetic position. In addition, the role of CPA members in plant tolerance to various abiotic stresses is summarized, and the future priority directions for CPA studies in plants are discussed.

## Introduction

During the process of evolution plants have evolved specific strategies and mechanisms to maintain ion homeostasis, regulate pH and adapt to continuously changing environmental conditions. Ion homeostasis is an essential part of plant life developmental cycle and resilience to a hostile environment. Plant membrane transporters play a critical role in these processes. It is estimated that around 18% of the predicted proteins of the *Arabidopsis* genome might contain two or more transmembrane domains (Ward, 2001; Schwacke et al., 2003). In addition, it is suggested that more than half of predicted membrane proteins could perform transporter functions (Tang et al., 2020). Many of them are key players in regulation of plant stress response to abiotic stresses such as salinity and drought (Yamaguchi et al., 2013; Hamamoto et al., 2015). Moreover, plants growth and development require uptake of essential minerals, and intracellular compartmentation and tissue-specific distribution of particular ions. For example, a proper functioning of stomata relies on operation of K^+^ and Cl^-^ channels and transporters in guard cells to modulate cell turgor for stomata opening and closure (Jezek and Blatt, 2017). Development of plant reproductive organs also requires coordinated work of membrane ion transport system (Sze et al., 2004). Another example is plant adaptive responses to salinity. Plant salt stress tolerance is strongly dependent on osmotic adjustment, exclusion of toxic ions from uptake, and their efficient intracellular compartmentation. All these three components of salt tolerance require involvement of various membrane transporters mediating water and ion transport across cellular membranes (Zhao et al., 2020).

Monovalent cation-proton antiporters (CPA) represent an important family of plant membrane transporters involved in various process during plant life cycle. According to the most recent classification (Saier et al., 2016), CPA transporters are divided into two major superfamilies, CPA1 and CPA2. The CPA1 superfamily is comprised of NHE/NHX (Na^+^/H^+^ exchanger, intracellular NHX) and NhaP/SOS1 (Na^+^/H^+^ antiporter, plasma membrane-bound NHX) subfamilies. These two subfamilies are found in a broad range of life systems including bacteria, fungi, metazoa, mammals and plants. The CPA2 family includes K^+^ Efflux Antiporter (KEA) and Cation/H^+^ Exchanger (CHX) subfamilies. Based on the current classification CPA2 family comprises bacterial, fungal, and plant transporters, with only one exception, namely human transmembrane and coiled-coil domain 3 (TMCO3; Saier et al., 2016). While CPA1 transporters received a substantial attention in the literature (mainly in the context of pH homeostasis and plant adaptive responses to salinity), many important aspects of their regulation remain elusive. The functional roles of plant membrane transporters belonging to CPA2 family are know much less.

The purpose of this review was to summarize the current knowledge about functions and physiological roles of CPA transporters in plants, and understand evolutionary relationships of NHE, NhaP, KEA, and CHX transporters from various plant species, in the context of their adaptation to hostile environments. We conclude that studies of cation/proton antiporters have to be prioritized in order to understand molecular mechanisms of salt tolerance, pH and mineral ion homeostasis, and plant growth and development under adverse environmental conditions.

## CPA1 Superfamily

**Figure 1:**
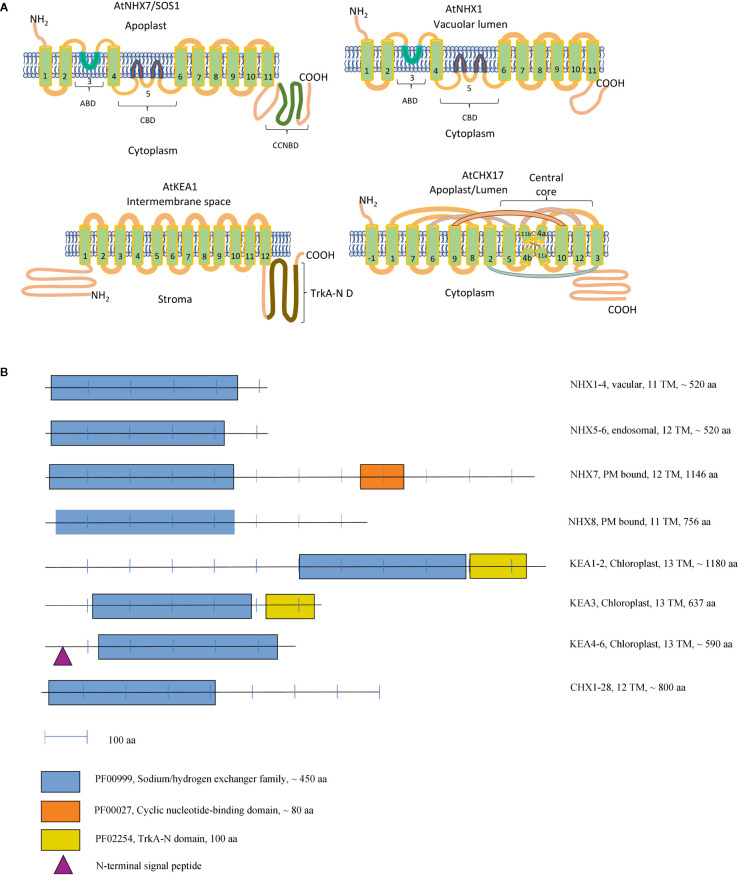
Topology Models of CPA Transporters **(A)**. A schematic representation of the domain organisation of the CPA proteins in Arabidopsis **(B)**. **(A)** ABD, Amiloride-binding domain; CBD, Cation-binding domain; CNBD, Cyclic nucleotide-binding domain; TrkA-N D, TrkA-N domain. KEA1 topology was constructed according to Bölter et al., 2019 and CHX17 topology according to Czerny et al., 2016. Extra TM of CHX17 seen at N terminus is labeled as −1. NHX1 and NHX7 topology was constructed according to Yamaguchi et al., 2003. For an alternative topology NHX model please see Sato and Sakaguchi, 2005. **(B)** Functional domains were identified according to Pfam database (El-Gebali et al., 2019), the number of the transmembrane domains and presence of the signaling peptide have been extracted from the ARAMEMNON database (Schwacke et al., 2003) (http://aramemnon.uni-koeln.de/index.ep).

CPA 1 superfamily includes two subfamilies of NhaP (also known as SOS) and NHE (known as NHX) transporters. Physiologically, both types of transporters operate as cation/H^+^ exchangers and have between 11 and 12 transmembrane domains (**Figure 1**). The members of this superfamily are relatively well characterised in terms of their physiological roles. However, the new experimental data suggest a much wider range of biological functions for these transport proteins as initially believed. Also, some novel aspects of their regulation have recently emerged.

### NHE/NHX Subfamily

**Figure 2:**
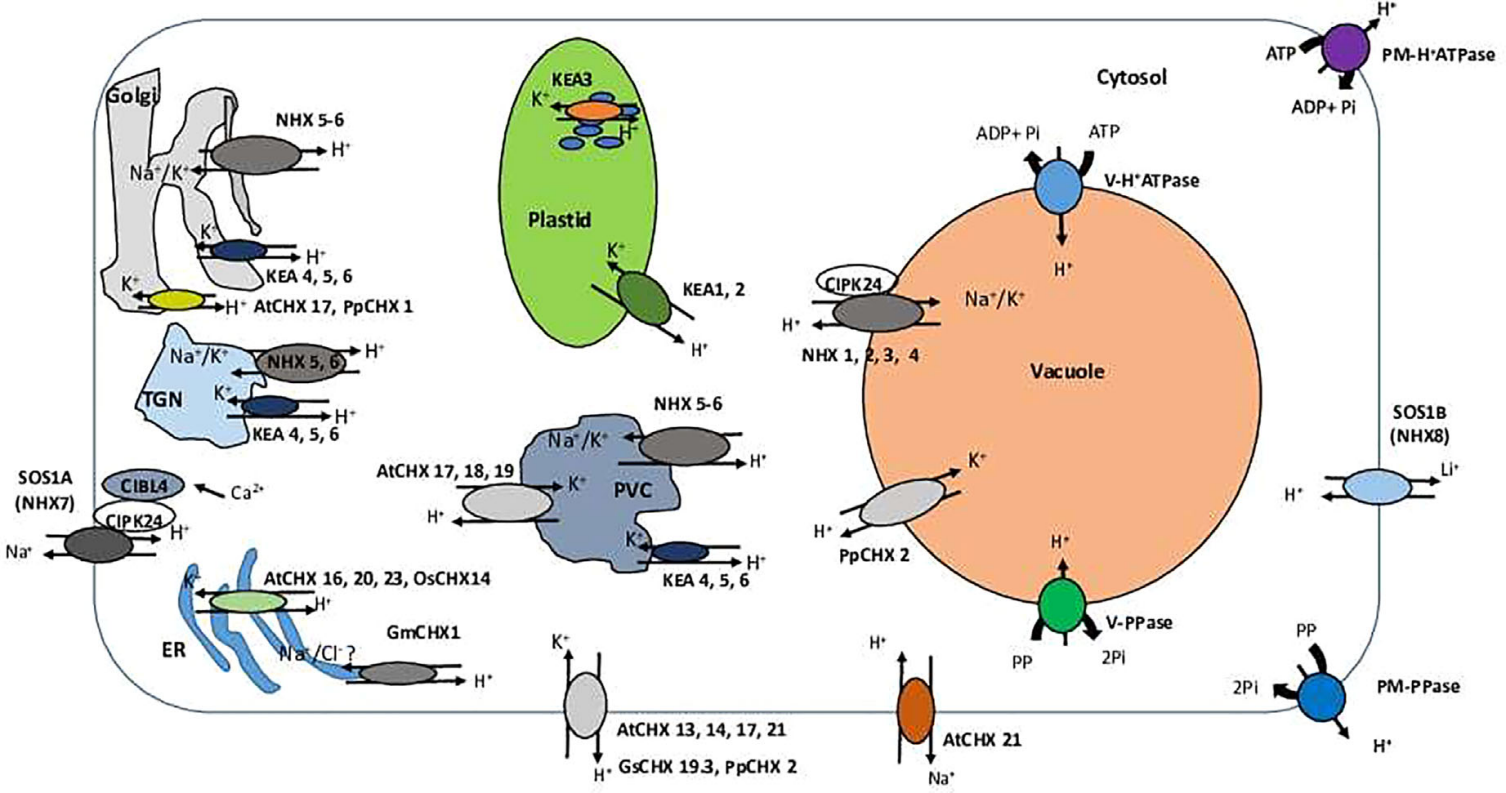
A schematic overview of the cellular localization and specialization of CPA family members. TGN, trans-Golgi network; PVC, prevacuolar compartment; ER, Endoplasmic reticulum; PM, plasma membrane; CHX, cation/H^+^ exchanger; KEA, K^+^-efflux antiporter; NHX (NHE), sodium proton exchanger; SOS (NhaP)- Salt Overly Sensitive; PM-H^+^ATPase, plasma membrane H^+^ATPase; V-H^+^ATPase, vacuolar H^+^ATPase; V-H^+^ATPase, vacuolar P H^+^ATPase; V-PPase, vacuolar PPase. SOS (NhaP) transporters are localized at the plasma membrane (PM) and involved in Na^+^ (SOS1A/NHX7) or Li^+^ (SOS1B/NHX8) removal from the cells. Activity of SOS1A/NHX7 is regulated by cytosolic Ca^2+^ *via* formation CIBL4-CIPK24 with SOS1B/NHX8 Na^+^ pump on the PM. CHX transporters AtCHX 13, 14, 17, 21 (*Arabidopsis thaliana*), GsCHX 19.3 (*Glycine soja*) and PpCHX 2 (*Physcomitrella patens*) are found to be localized on the PM and involved in a cellular K^+^ uptake in exchange to H^+^. AtCHX 21 may also participate in Na^+^ removal from the cells and their loading into the xylem. NHX1-4 exchangers are localized at the tonoplast of the central vacuole and involved in K^+^ or Na^+^ vacuolar sequestration. NHX5-6 is present at Golgi, TGN, and PVC membranes and participate in K^+^ or Na^+^ sequestration into endosomal lumen. KEA 4–6 demonstrate the same pattern of cellular localization and involved in K^+^ transport into endosomal lumen. In addition, AtCHX 17, 18, 19 are targeted to PVC membrane and, very likely, are involved in K^+^ sequestration in this organelles. AtCHX 17 and PpCHX 1 were found to be localized at Golgi membranes and transport K^+^ to Golgi lumen (and ). Arabidopsis AtCHX 16, 20, 23, and rice OsCHX14 have ER localization and operate in K^+^ sequestration in the ER lumen. The similar localization pattern was found for the soybean GmCHX1; however, this exchanger is possibly responsible for transports of Na^+^ and Cl^-^ instead. KEA 1-3 exhibit plastid localization. KEA 1 and 2 are localized at the inner envelope plastid membrane, while KEA 3 is targeted to the thylakoid membrane. All plastidial KEA members are mainly involved in K^+^ transport and accumulation.

NHX antiporters catalyse the electroneutral exchange of H^+^ for Na^+^ or K^+^. Eight NHX isoforms are present in Arabidopsis genome. Four of these (AtNHX1 to AtNHX4) are located at the tonoplast; two (AtNHX7 and AtNHX8) at the plasma membrane; and two (AtNHX5 and AtNHX6) are localized in endosomes (the Golgi, trans-Golgi, and late endosomes/prevacuolar compartments) (**Figure 2**) (Bassil et al., 2011a; Bassil et al., 2011b; McCubbin et al., 2014; Reguera et al., 2015). Initially described as Na^+^/H^+^ antiporters involved in vacuolar Na^+^ sequestration (Apse et al., 1999), NHX exchangers were later shown to also have high affinity to K^+^ (Bassil and Blumwald, 2014). As a result, depending on their intracellular localization, NHX exchangers are capable to transport either K^+^ or Na^+^ into the vacuole or endosome in exchange for H^+^ efflux to the cytosol (NHX1–6) and Na^+^ efflux out of the cell in exchange for H^+^ influx into the cell (plasma membrane-bound NHX7–8) (Jiang et al., 2010). The predominant isoforms are AtNHX1 and AtNHX2, found in roots, shoots and seedlings; expression levels of AtNHX3, 4, and 6 are much lower in these tissues (Rodriguez-Rosales et al., 2009).

The hydropathy analysis of plant NHX indicates a domain organization similar to NHE isoforms, suggesting that structural features are conserved across the families (Rodriguez-Rosales et al., 2009). However, no consensus exists about the topology of plant NHX transporters. According to one of the models, AtNHX1 topology closely resembles that for human NHE protein, with 11 transmembrane helices in the conserved hydrophobic N-terminal domain, and a loop corresponding to hydrophobic region 9 (Sato and Sakaguchi, 2005). The first transmembrane helix of AtNHX1, corresponding to transmembrane helix 2 in NHE1, is inserted in the same orientation into the membrane, whilst the C-terminus is exposed to the cytoplasm involved in regulatory interactions (Sato and Sakaguchi, 2005). Another model implies that the C-terminal domain would be exposed to the vacuolar lumen, whilst the N-terminus would be cytoplasmic (**Figure 1**) (Yamaguchi et al., 2003). This model predicts only 9 transmembrane helices, hydrophobic domain 3, containing the putative amiloride binding domain, and the hydrophobic domains 5 and 6, containing residues that are likely involved in Na^+^ or H^+^ binding and transport, would not cross the membrane. The physiological rationale of such arrangement is that such structure would result in several transmembrane helices being inserted in the opposite direction in the membrane, thus allowing NHX operate as an antiporter. Both models are tentative now and require a full and independent validation.

**Figure 3:**
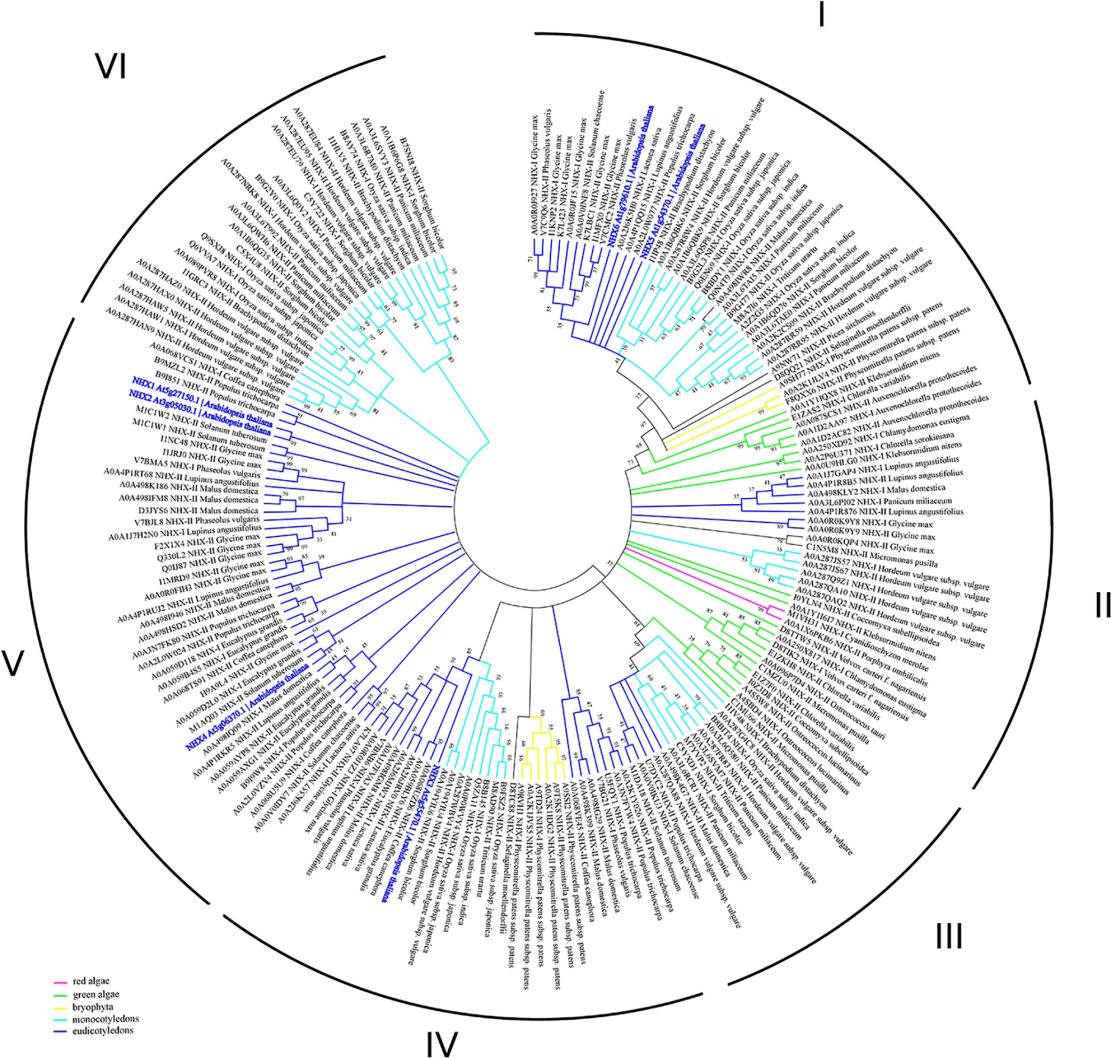
Phylogenetic analysis of NHX proteins. The Maximum Likelihood method and “Dayhoff” model were used; 1,000 bootstrap replicates. The phylogenetic tree was obtained with MEGA X software on the basis of full-length protein sequences. Branches colours are: magenta, red algae; green, green algae; yellow, bryophyta; light blue, monocotyledons; blue, eudicotyledons. Arabidopsis thaliana sequences are highlighted in bold blue. The species used in this study are listed in **Supplementary Table 1**. NHX1-8, KEA1-6 and CHX1-28 proteins from *Arabidopsis thaliana* were used for the BLAST-based identification of the corresponding transporters (BLASTp tool—Shiryev et al., 2007). The obtained proteins (named with Uniprot ID) were aligned with MUSCLE (Edgar, 2004) implemented in Ugene software (Okonechnikov et al., 2012). Pfam database (El-Gebali et al., 2019) was used for the identification of the domain. MEGA X software was used for the phylogenetic analysis. A comparison of the structural models is presented in **Figure 1B**.

Previous studies suggested that the NHE/NHX family emerges from the diverged cyanobacterial NhaP gene (Brett et al., 2005). According to this data, together with NhaP/SOS1 proteins, NHXs transporters are distributed between three different clades (Chanroj et al., 2012). However, NHX family has passed through several duplication events and is represented by several proteins in every species. According to our phylogenetic analysis, the clade diversity of NHX transporters is much wider (**Figure 3**, **Table S1**). Based on intercellular localization of NHE/NHX, our phylogenetic analysis suggests the presence of six clades of the NHX proteins (**Figure 3**). Interestingly, NHX members form green algae (*Auxenochlorella protothecoides*, *Chlorella variabilis*, and *Klebsormidium nitens*) and primitive vascular plants such as a moss *Physcomitrella patens* exhibit closest relation to the endosomal NHX5-6 members. Both clades I and II are the most complex, being comprised of the members from red and green algae, Bryophyta, and mono- and dicotyledon species. In general, green algae proteins appear in I, II, and III clades. On the contrary, clades V and VI are discrete and formed by only dicotyledon and monocotyledon species, respectively. Based on results of our phylogenetic analysis it may be suggested that NHE/NHX family have passed a gradual evolutionary complication from clade I to VI. The most primitive clades I-III include a wide range of proteins from studied taxa and cluster with NHX5-6. Clades V and VI represent the most recent and specialized proteins, with only mono- and dicotyledon species included, and are related to the vacuolar NHX proteins from *Arabidopsis*. Clade IV represents a transitional clade, which also includes, in addition to the mono- and dicotyledon species, *Physcomitrella patens* proteins (**Figure 3**). Thus, according to our phylogenetic analysis the phylogenetic diversity NHE/NHX family members is much wider than suggested by previous studies and comprises 6 different clades, not three as believed (Chanroj et al., 2012). Our data also suggests that vacuolar NHX specialization is one of most recent evolutional events. It is very likely that “colonisation” of the vacuolar tonoplast membrane by NHE/NHX transporters provided sufficient advantages for higher plants to resist various environmental challenges for survival on land.

The activity of animal NHE proteins can be regulated by a variety of regulatory mechanisms involving the long C-terminal tail (Rodriguez-Rosales et al., 2009). In plants, removal of the last 82 amino acids in AtNHX1 protein modified its selectivity and increased its preference for Na^+^/H^+^ exchange (Yamaguchi et al., 2003). It was also shown that C-terminal domain interacts with a CaM-Like protein AtCaM15 (Yamaguchi et al., 2005), with CaM15 binding inhibiting Na^+^/H^+^ antiport and increasing NHX1 specificity for K^+^. AtNHX1 activity could be also regulated through interaction with the protein kinase SOS2 (Qiu et al., 2002).

Given such diversity and a broad spectrum of functions, NHX exchangers are believed to be central to both developmental and adaptive responses in plants. Indeed, onset of hypoxia in flooded soils results in a sharp decrease in cytosolic pH (by 0.5 to 0.6 pH units) within minutes or even seconds of stress onset (Ratcliffe, 1997; Felle, 2005), and vacuolar NHXs are firmly associated with intracellular pH regulation (Bassil et al., 2012). The initial cloning and overexpression of AtNHX1 in Arabidopsis firmly demonstrated its important role in salinity stress tolerance (Apse et al., 1999), and many additional studies subsequently confirmed that NHX overexpression lead to improved salt tolerance in diverse species (Zhang and Blumwald, 2001; Ohta et al., 2002; Rodriguez-Rosales et al., 2008). NHX transcript levels are induced by salt in both leaf (Quintero et al., 2000; Venema et al., 2003; Kagami and Suzuki, 2005; Li et al., 2018; Sun et al., 2019; Su et al., 2020) and roots (Fukuda et al., 2004a; Fukuda et al., 2004b; Zörb et al., 2004; Ye et al., 2009; Zhang et al., 2018). A similar increase was reported upon plant exposure to dehydration (Li et al., 2006), hyperosmotic stress (Fukuda et al., 2004a; Fukuda et al., 2004b Yokoi et al., 2002), or ABA treatment (Yokoi et al., 2002; Venema et al., 2003). Recent analysis of NHX1 orthologous regions in rice revealed that Oryza AA genomes cluster distinctly from NHX1 regions from more ancestral *Oryza* BB, FF, and KKLL genomes (Sellamuthu et al., 2020). These authors have also reported the presence of a retro-copy of the OcNHX1 cDNA in the genome of halophytic *O. coarctata* wild rice (rOcNHX1), linking intron retention, and splicing events of this gene with evolution of salinity tolerance.

Another hallmark of salinity stress is a massive K^+^ leak from the cell (Shabala and Pottosin, 2014; Wu et al., 2018; Rubio et al., 2020) resulting from Na-induced depolarization of plasma membrane and increased permeability of outward-rectifying depolarization-activated K^+^ channels (Shabala and Cuin, 2008; Shabala et al., 2016). While this K^+^ loss may be an important “master switch” re-programming cell from energy-consuming biosynthesis towards defence/reparation needs (Demidchik, 2014; Shabala, 2017; Rubio et al., 2020), reduced cytosolic K^+^ concentrations may also activate caspase-like proteases and endonucleases resulting in programmed cell death (Shabala, 2009; Demidchik et al., 2010). Thus, maintenance of optimal cytosolic K^+^ levels seems is absolutely essential for plants survival under saline conditions. Given high affinity of some NHX isoforms towards K^+^, the role of NHX exchangers in regulation of cytosolic K^+^ levels is beyond any doubt. Also, the ability of NHX to pump both Na^+^ and K^+^ into vacuole contributes to maintenance of cell turgor pressure and may be essential to help plants to cope with drought conditions (Barragán et al., 2012). NHX operations were also shown to be essential for stomatal regulation (Andrés et al., 2014) and flower development (Yoshida et al., 2005; Bassil et al., 2011b). NHX transporters also function in plant tolerance to Al^3+^ toxicity. Ectopic expression of *HtNHX1* from *Helianthus tuberosus* in rice enhanced Al^3+^-trigged-secretion of citrate acids and rhizosphere acidification, as well as reduced K^+^ efflux from root tissues (Li et al., 2020) while expression of *HtNHX2* prevented Al^3+^- trigged-decrease of H^+^ influx into root tissues.

### NhaP Subfamily

The NhaP subfamily originates from ancestral NhaP genes in prokaryotes and known to transport Na^+^ or Li^+^ in exchange for H^+^ in an electroneutral and pH-dependent manner (An et al., 2007). The model plant Arabidopsis has two NhaP homologs At2g01980 (SOS1A) and At1g14660 (SOS1B), also called NHX7 and NHX8 by some authors (Li et al., 2010). Similarly to the NHX subfamily, NhaP proteins have the same Sodium/Hydrogen exchanger domain (Pfam database domain PF00999; El-Gebali et al., 2019) but is rather different on the amino acid level (Pires et al., 2013). Indeed, recent phylogenetic studies have defined a rather distinct relation between NhaP subfamily and NHX family and independent evolutionary pathway (Brett et al., 2005; Pires et al., 2013). In comparison to the NHX family, the number of SOS1 genes in eukaryote genomes is limited to only a few copies (Chanroj et al., 2012; Oh et al., 2013). Based on the sequence similarity, NhaP homologs could be identified in all known photosynthetic species (Pires et al., 2013). Despite the impressive number of experimental studies dissecting physiological functions of plant NhaPs, many aspects of SOS1A and SOS1B regulation and functionality remain unclear.

One of the main features of the SOS-like proteins identified in plants is the presence of the long C-terminal tail, required for its operation (**Figure 1**). The C-terminus tail has an auto-inhibitory C-terminal domain, phosphorylation sites and is required for the protein-protein interaction (Quintero et al., 2011) and dimerization (Ullah et al., 2016). The main difference between SOS1A and SOS1B is likely to be in the presence of the cyclic nucleotide-binding domain (PF00027), located in the middle of the C-terminus tail (**Figure 1B**). Such cyclic nucleotide-binding domains are known to bind to the wide range of protein targets, in order to regulate multiple cellular processes (Shabb and Corbin, 1992; LaFranzo et al., 2010; Rehmann et al., 2017; VanSchouwen et al., 2017). SOS1A and SOS1B are both localized to the plasma membrane (Shi et al., 2000; An et al., 2007) (**Figure 2**). There is also a difference in their selectivity. While SOS1A is a Na^+^/H^+^ antiporter (Guo et al., 2009), SOS1B seems to only transport Li^+^ (An et al., 2007) (**Figure 2**).

The key functional role of SOS1A is in the extrusion of the excessive Na^+^ out of the cells thus preventing Na^+^ toxicity in the cytosol. This extrusion occurs at two critical points: 1) in epidermal root cells (e.g. extruding Na^+^ back into the rhizosphere), or 2) at the xylem/parenchyma interface, loading Na^+^ into the xylem (Shi et al., 2000; Zhao et al., 2020). SOS1 is autoinhibited under normal conditions, and this inhibition is released by the phosphorylation of Ser1044 at the C-terminal domain of SOS1A by SOS2 (Quintero et al., 2011), a serine/threonine protein kinase belonging to the sucrose non-fermenting 1 (SNF1)/AMPK family (Halfter et al., 2000). In its turn, SOS2 is activated and recruited to the plasma membrane *via* its interaction with SOS3, a calcineurin B-like protein (encoded by AtCBL4 in Arabidopsis). Collectively, this process is known as a SOS signaling pathway. Under normal conditions, the kinase activity of SOS2 is inhibited by 14-3-3 and GIGANTEA (GI) proteins (Zhao et al., 2020). When plants are exposed to salinity, 14-3-3 and GI are degraded, and SOS2 is released from SOS2-GI/14-3-3 complexes (Kim et al., 2013; Zhou et al., 2014). As a result, SOS1A-mediated Na^+^/H^+^ exchanger operation is activated. The kinase activity of SOS2 is also regulated by the protein phosphatase 2C ABI2 (Ohta et al., 2003).

A recent study has shown that another calcineurin B-like protein (AtCBL10; At4g33000) could mediate Ca^2+-^dependent salt stress response (Yang et al., 2019) by forming a complex with SOS2 and activating SOS1A; this activation is independent of (and complimentary to) SOS3 (AtCBL4)– dependent activation. Also, CBL10 could participate in a vacuolar Na^+^ homeostasis and interact with other protein kinases (Kim et al., 2007; Waadt et al., 2008). Direct interaction with TOC34 translocon at the outer envelope membrane of chloroplasts 34) allows CBL10 to modulate Ca^2+^ signals in diverse ways (Cho et al., 2016). In addition, salt stress-induced SOS1A/SOS2 complex interacts, phosphorylates and activates PUT3 polyamine transporter (Chai et al., 2020), leading to accumulation of polyamine in the cytosol and protecting cells from oxidative stress (Shen et al., 2016). Polyamines are also potential blockers of tonoplast slow (SV) vacuolar channels (Pottosin et al., 2014). This “locks” the salt cargo in the vacuole and prevents Na^+^ back-leak into cytosol, thus preventing futile cycle at the tonoplast (Shabala et al., 2020). The above trait is crucial for salinity tissue tolerance, in the context of the overall metabolic energy cost of adaptation (Munns et al., 2020). As PUT3 activity is pH-dependent, changes in cytosolic pH resulting from SOS1A operation favour PUT3 transporting activity (Fujita and Shinozaki, 2014).

Another SOS1A mediated salt stress response is closely related to regulatory RCD1 (RADICAL‐INDUCED CELL DEATH1) element. RCD1 (RADICAL‐INDUCED CELL DEATH1) is a hub gene, combining and regulating several stress-related and development pathways *via* interaction with multiple transcription factors (Jaspers et al., 2009; O’Shea et al., 2015). Normally, RCD1 is localized to the nucleus but in case of salt and oxidative stress, RCD1 was also found in the cytoplasm. It was predicted that RCD1 could interact with C-terminal tail of SOS1A in the cytoplasm (Katiyar-Agarwal et al., 2006). SOS1-RCD1 interaction mediates a functional cross-talk between ion homeostasis and ROS detoxification systems.

The SOS1B was subjected to the functional characterisation and involvement in the plant’s stress-response pathways (An et al., 2007) and shown to act mainly as a Li^+^ and K^+^ transporter. Detailed analyses of the SOS1B polymorphism variants in 20 Arabidopsis have defined recent adaptive changes facilitating salt-stress response, unique among other stress-related genes (Puerma and Aguadé, 2013).

**Figure 4:**
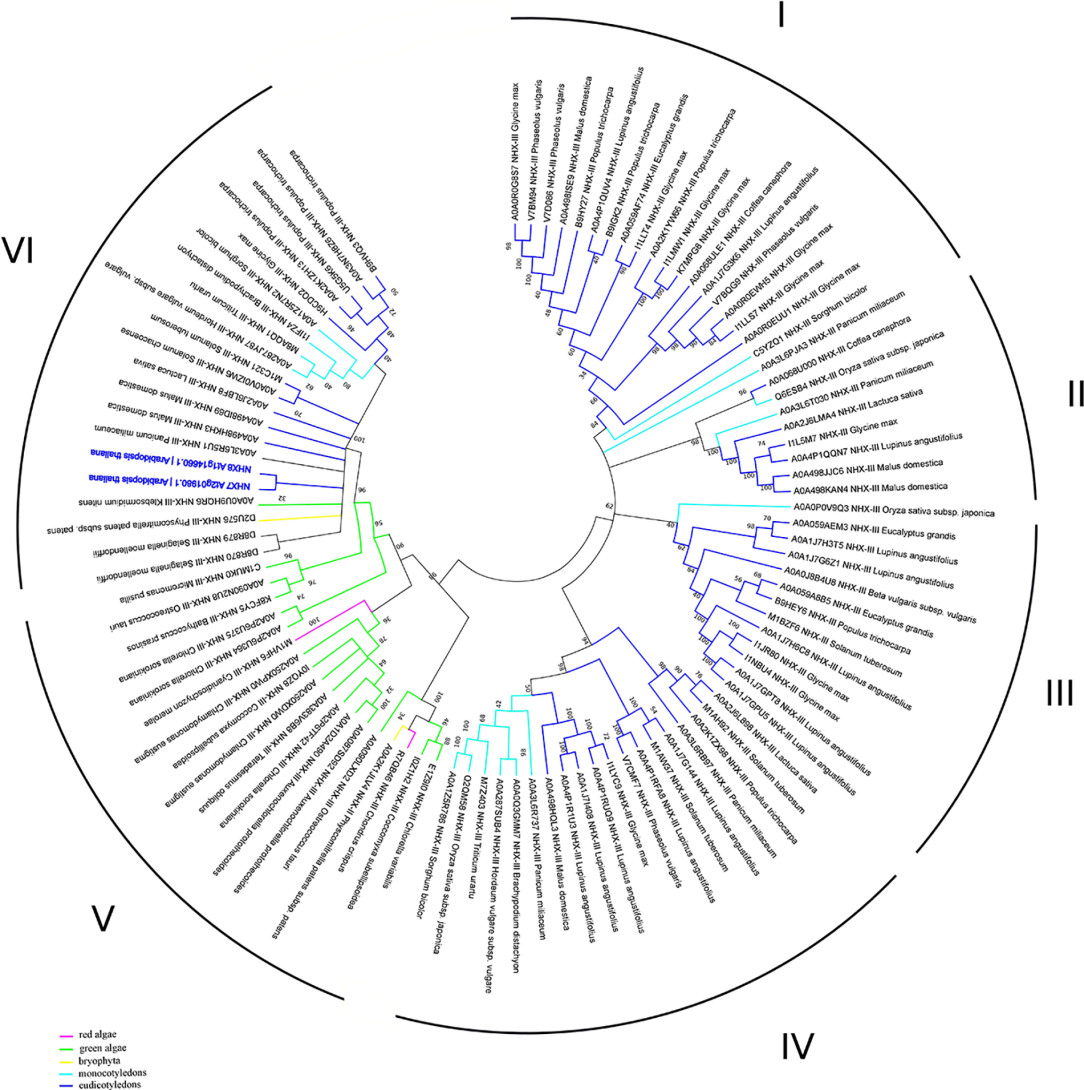
Phylogenetic analysis of NhaP proteins. The Maximum Likelihood method and “Dayhoff” model were used; 1,000 bootstrap replicates. The phylogenetic tree was obtained with MEGA X software on the basis of full-length protein sequences. Branches colours are: magenta, red algae; green, green algae; yellow, bryophyta; light blue, monocotyledons; blue, eudicotyledons. *Arabidopsis thaliana* sequences are highlighted in bold blue.

In addition to the prokaryotic origin of the SOS1 proteins in the green lineage (Brett et al., 2005), phylogenetic studies have defined fewer recent duplication events in comparison to the NHX family members. It is plausible to suggest that the recent SOS1 duplication could lead to the emerging of the SOS1B form, thus broadening plant’s ability to tolerate adverse environmental conditions (Pires et al., 2013). Until now, NhaP family was subdivided into three clades: monocot, dicot and moss/spikemoss (Chanroj et al., 2012; Pires et al., 2013). Results of our bioinformatic analyses, however, have also revealed the presence of the NhaP family members in green algae, thus demonstrating the presence of the 6 clades, suggesting more complex phylogenetic diversity of NhaP family in plants. Interestingly, green algae have both SOS1A and SOS1B homologs (e.g. as in *Chlorella sorokiniana;* **Figure 4**). They also suggest that the NhaP family was formed by the recent duplication and specialization events. Also, while clades I–IV are formed only by mono- and dicotyledon species, clade V includes only algae and bryophyte (**Figure 4**, **Table S1**). Clade VI represents, probably, the most specialized group of proteins and includes also marker sequences from *Arabidopsis thaliana* (**Figure 4**, **Table S1**). Thus, that combination of functional domains (sodium/hydrogen exchanger + cyclic nucleotide-binding) allowing to recognize NHX7/SOS1A homologues is rather unique and not present in bacteria. Therefore, this combination of SOS1A functional domains exhibits high specificity for the green lineage. Based on this analysis, it appears that SOS1 homologues in their modern form (sodium/hydrogen exchanger domain followed by the cyclic nucleotide-binding domain) were first formed in algae and then appeared in land plants.

## CPA2 Superfamily

CPA 2 superfamily is comprised of K^+^-efflux antiporter (KEA) and cation/H^+^ exchanger (CHX) subfamilies. Both subfamily types have from eight to 14 transmembrane domains (TMs) in their structure (Chanroj et al., 2012). Although the CPA2 family represent a large number of identified proteins, the physiological and function for the majority of them still need to be characterized.

### KEA Subfamily

**Figure 5:**
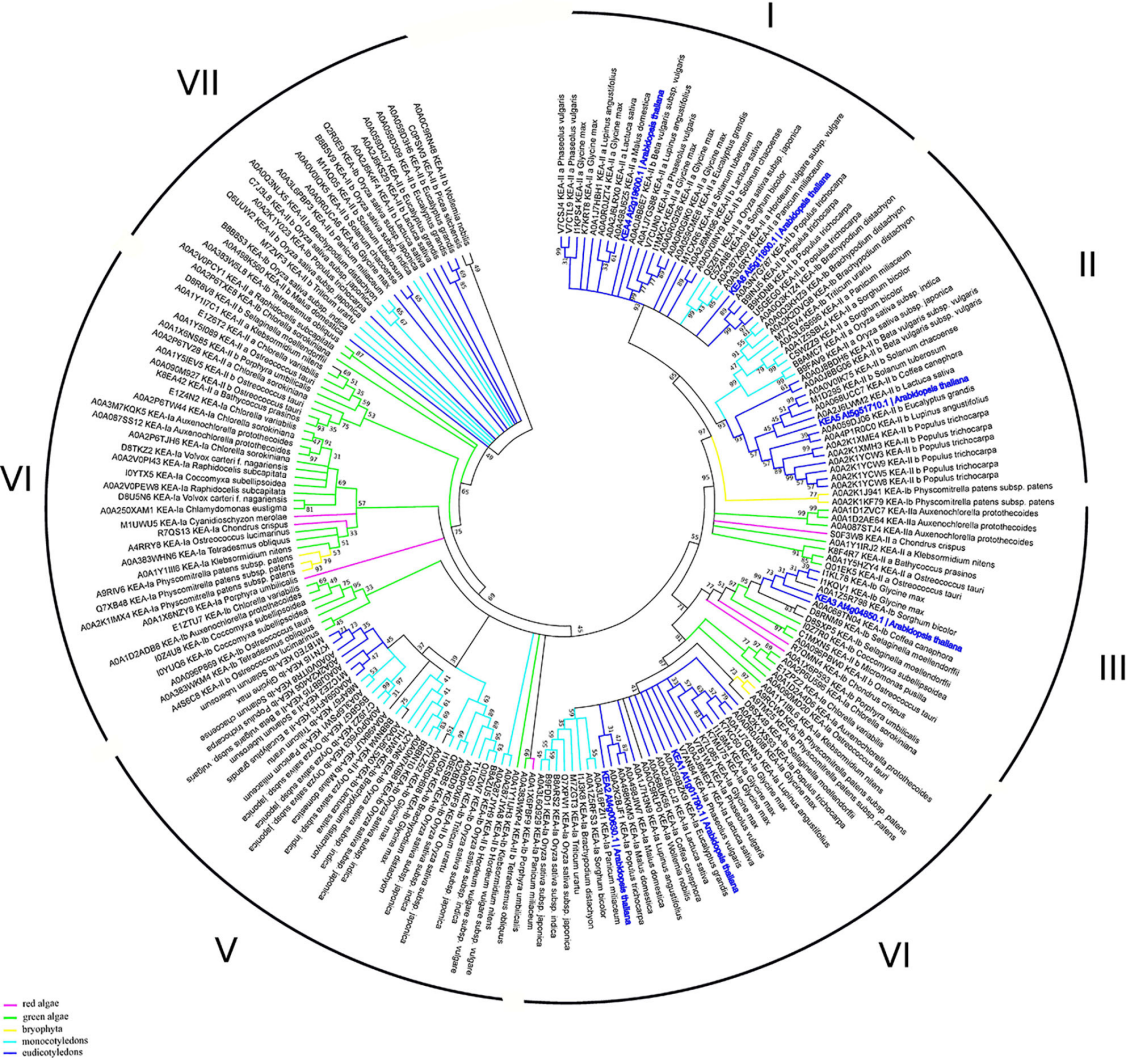
Phylogenetic analysis of KEA proteins. The Maximum Likelihood method and “Dayhoff” model were used; 1,000 bootstrap replicates. The phylogenetic tree was obtained with MEGA X software on the basis of full-length protein sequences. Branches colours are: magenta, red algae; green, green algae; yellow, bryophyta; light blue, monocotyledons; blue, eudicotyledons. *Arabidopsis thaliana* sequences are highlighted in bold blue.

The K^+^ efflux antiporter (KEA) proteins of plants show homology to the bacterial K^+^/H^+^ antiporters KefC and KefB, comprised of a soluble N-terminal Na_H exchanger domain and a C-terminal KTN NAD(H)-binding domain (Chanroj et al., 2012). A phylogenetic analysis divides the KEAs into two clades, KEAI and KEAII. According to the properties of its N-terminus, KEAI was further divided into two groups: KEAIa, with a long soluble N-terminal domain of about 570–770 amino acids, and KEAIb, without the N-terminal extension (Chanroj et al., 2012). Our analysis of KEA family domain architecture suggest that these transporters could be further sub-divided to the three groups: 1) KEA1-2, with domain-free N-terminal tail, central sodium/hydrogen exchanger (PF00999) and C-terminal TrkA-N domains (PF02254); 2) KEA3—sodium/hydrogen exchanger and C-terminal TrkA-N domains; and (3) KEA4-6, with only sodium/hydrogen exchanger domain (**Figure 1**). It was predicted that only KEA4-6 has an N-terminal signal peptide. The TrkA-N domain is a NAD-binding (Zhang et al., 2020) that is required for the proper function of many transporters, including potassium channels (Jiang et al., 2002). Interestingly, the results of our phylogenetic study suggest the presence of seven clades of the KEA proteins (only two were described in by Chanroj et al., 2012) (**Figure 5**). The high level of sequence similarities may indicate a close evolutional relation between proteins. Interestingly, the distribution of marker proteins (*Arabidopsis thaliana*—highlighted in bold blue) on the phylogenetic tree is well-correlated with their subcellular localization and a presence of the TrkA-N domain. Thus, KEA 4, 5, and 6, currying predicted signaling peptide, are located in clade I and II, that include only mono- and dicotyledon species. On the contrary, KEA 1, 2, and 3 are predicted to be located in chloroplasts and mitochondria, appear in clades III and IV with all studied taxa (red and green algae, bryophyte, mono- and dicotyledons) (**Figure 5**, **Table S1**).

Functional studies on yeast revealed that ability of six Arabidopsis AtKEAs to transport K^+^ (Zheng et al., 2013). Notably, the truncated AtsKEA1 and AtsKEA2 lacking its long N-terminus had the highest resistance to elevated external K^+^ among AtKEA family members. Moreover, AtsKEA2 had a cation/H^+^ exchange with preference for K^+^ = Cs^+^ > Li^+^ > Na^+^ (Aranda-Sicilia et al., 2012). In *E. coli* expression system, AtKEA1-3, and AtKEA5 showed a bi-directional K^+^ transport activity, whereas AtKEA4 and AtKEA6 functioned as a K^+^ uptake system (Tsujii et al., 2019). AtKEA1-6 were dominant for K^+^ transport, indicating that AtKEAs were involved in electric potential (ΔΨ) formation (Zheng et al., 2013; Tsujii et al., 2019).

The *AtKEAs* genes were widely expressed throughout plant growth and development (Zheng et al., 2013; Han et al., 2015) (**Table 1**). GUS staining exhibited overlapping expression patterns for AtKEAs, with only some subtle differences (Han et al., 2015; Zhu et al., 2018). Besides, *AtKEAs* expression was also responsive to environmental stress, such as K^+^ deficiency, salt and osmotic stress (Zheng et al., 2013; Han et al., 2015; Jia et al., 2018) (**Table 1**).

**Table 1:** Selected examples of the properties, tissue-specific expression, and physiological functions of KEA transporters in plants.

KEA isoforms	Clade	Tissueexpression	Membranelocalization	Transportproperties	Demonstratedfunction	References
In *Arabidopsis thaliana*						
AtKEA1	IV	Mainly in shoots, leaf veins, hypocotyls; sepals, filaments, pollen, stigmas	Inner envelope membrane of plastids	K^+^	Plastid division and thylakoid membrane formation; rapid hyperosmotic-induced Ca^2+^ responses; upregulation by K^+^ deficiency and salinity.	Zheng et al., 2013; Kunz et al., 2014; Han et al., 2015; Aranda-Sicilia et al., 2016; Stephan et al., 2016; Tsujii et al., 2019
AtKEA2	IV	Ubiquitous, root steles, leaf veins; sepals and filaments	Inner envelope membrane of plastids	K^+^ = Cs^+^ > Li^+^ > Na^+^	Plastid division and thylakoid membrane formation; rapid hyperosmotic-induced Ca^2+^ responses; upregulation by K^+^ deficiency, salinity and osmotic stress.	Aranda-Sicilia et al., 2012; Zheng et al., 2013; Kunz et al., 2014; Han et al., 2015; Aranda-Sicilia et al., 2016; Stephan et al., 2016; Tsujii et al., 2019
AtKEA3	III	Mainly in shoots, leaf veins, sepals, filaments and stigmas	Thylakoid membrane	K^+^	Chloroplast proton motive force; rapid hyperosmotic-induced Ca^2+^ responses; upregulation by K^+^ deficiency and salinity.	Zheng et al., 2013; Armbruster et al., 2014; Kunz et al., 2014; Han et al., 2015; Stephan et al., 2016; Armbruster et al., 2017; Dukic et al., 2019; Tsujii et al., 2019
AtKEA4	I	Mainly in roots, root tips and steles, root hairs; sepals, filaments and pollen	Golgi, TGN and PVC/MVB, mainly at the Golgi	K^+^	pH and K^+^ homeostasis in endomembrane compartments; upregulation by K^+^ deficiency.	Zheng et al., 2013; Han et al., 2015; Zhu et al., 2018; Tsujii et al., 2019; Wang et al., 2019
AtKEA5	II	Steles of the mature roots, root hairs; sepals, filaments, and pollen	Golgi, TGN and PVC/MVB, mainly at the Golgi	K^+^	pH and K^+^ homeostasis in endomembrane compartments; upregulation by K^+^ deficiency; salinity and osmotic stress.	Zheng et al., 2013; Han et al., 2015; Zhu et al., 2018; Tsujii et al., 2019; Wang et al., 2019
AtKEA6	I	Hypocotyls and roots; sepals and pollen	Golgi, TGN and PVC/MVB, mainly at the Golgi	K^+^	pH and K^+^ homeostasis in endomembrane compartments; upregulation by sucrose treatment in the shoots.	Zheng et al., 2013; Han et al., 2015; Zhu et al., 2018; Tsujii et al., 2019; Wang et al., 2019
In *Oryza sativa* L.						
AM1/CDE3 (Os04g58620)	IV	Mainly in leaves	Chloroplasts		Chloroplast development and drought tolerance; upregulation by salt and PEG.	Sheng et al., 2014; Luo et al., 2018
In *Glycine max* L.						
Glyma16g32821 (GmKEA5)	II	Ubiquitous	PM?		Upregulation by salt and low K^+^ treatment.	Chen et al., 2015
Glyma18g46420 and Glyma09g39770 (GmKEA2)	IV	Ubiquitous	PM (predicted)		Nodulation; up regulated in early time and down regulated later in root under low K^+^, high Na^+^ and K^+^ stress.	Chen et al., 2015; Rehman et al., 2017

The reverse genetic and proteomic studies have shown that AtKEA1-3 function in the plastid (**Table 1**, **Figure 1B**). AtKEA1 and AtKEA2 are targeted to the inner envelope membrane of chloroplasts, determined by their long N-terminal domain (Aranda-Sicilia et al., 2012; Aranda-Sicilia et al., 2016). The phenotypic analysis showed that *kea1 kea2* double mutant had retarded growth, fewer and swollen chloroplasts with disrupted envelope membranes and reduced thylakoid, but also delayed formation of pigments and electron transport complexes in pale green leaves (Kunz et al., 2014; Aranda-Sicilia et al., 2016). Furthermore, AtKEA1 and AtKEA2 specifically distribute to the polar sites in small and dividing plastids separated by the fission planes, where they might be involved in modulation of the specific inner envelope microdomains osmotic, ionic, and pH homeostasis; the processes that are important for plastid division and thylakoid membrane formation (Aranda-Sicilia et al., 2016) (**Figure 2**). A topology analysis indicated that both of N-terminal and C-terminal regulatory domains of AtKEA1(2) reside in the chloroplast stroma, as required for physiological functions (Bölter et al., 2019) (**Figure 1**). AtKEA3 is localized to the thylakoid membrane, mediating the light efficiency of photosynthesis in fluctuating light by modulating the proton gradient (ΔpH) and electric potential (ΔΨ) which contribute to proton motive force (pmf) (Armbruster et al., 2014; Kunz et al., 2014; Armbruster et al., 2017; Dukic et al., 2019) (**Table 1**). AtKEA3 activity might be regulated by a mechanism involving its C-terminus which contains a KTN domain (Armbruster et al., 2016) (**Figure 1**). A dominant mutant allele of KEA3, *disturbed proton gradient regulation (dpgr)*, showed reduced non-photochemical energy quenching (NPQ) during induction of photosynthesis (Wang et al., 2017). Overexpressing the DPGR-type KEA3 (DPGRox) inhibited plant growth, with disturbed ΔpH-dependent regulation of electron transport (Wang and Shikanai, 2019). It was recently shown that AtKEA3 benefited photosynthesis and growth in chloroplast ATP synthase-compromised mutant, *via* a KEA3-dependent reduction of ΔpH (Correa Galvis et al., 2020). In addition, AtKEA1-3 are necessary to generate rapid Ca^2+^ responses to hyperosmotic stimuli (Stephan et al., 2016).

Members of AtKEA4–6 group share similar subcellular localization, being distributed along the Golgi, trans-Golgi network (TGN) and the pre-vacuolar compartment/multivesicular bodies (PVC/MVB), but found mainly at the Golgi (Zhu et al., 2018; Wang et al., 2019) (**Figure 2**, **Table 1**). The loss of AtKEA4-6 function has a dramatic impact on pH and K^+^ homeostasis in endomembrane compartments, salt tolerance and plant growth (Zhu et al., 2018). Also, AtKEA4-6 functioned in cell wall biosynthesis during rapid etiolated seedling growth (Wang et al., 2019). Additionally, endosomal AtNHX5 and AtNHX6 partially recovered the defects of *kea4 kea5 kea6* triple mutant in endosomal pH homeostasis and salt tolerance, suggesting plant endosomes required the coordinated function of a larger number of K^+^/H^+^ exchangers, including KEA4, KEA5, KEA6, NHX5, and NHX6 (Sze and Chanroj, 2018; Zhu et al., 2018) (**Figure 2**).

Up to now, numerous physiological roles of KEAs in crops have also been reported (**Table 1**). In rice, *AM1* (*Albino midrib 1*)/*CDE3* (*Chlorophyll deficient 3*), encoding a K^+^ efflux antiporter protein, was involved in chloroplast development and drought tolerance (Sheng et al., 2014; Luo et al., 2018).

Based on the analysis of amino acid sequence, 12 *GmKEA* genes were identified in soybean, located in three branches in the phylogenetic tree, most of which expression were responsive to nodulation and abiotic stresses, such as K^+^ deficiency and salinity (Chen et al., 2015; Rehman et al., 2017). Some studies suggested that some GmKEAs could be localized to the plasma membrane (PM)—e.g. GmKEA2 (Glyma09g39770) and GmKEA5 (Glyma16g32821) (Chen et al., 2015; Rehman et al., 2017) (**Table 1**), although in the former case this unusual PM localization was only predicted (Rehman et al., 2017). For GmKEA5, the localization pattern was based on GFP-tagged GmKEA5 distribution in the transformed onion cells (Chen et al., 2015). However, reported results have several serious limitations. Firstly, the authors have used only C-terminal GFP fusion constructs under control 35 S promoter. It would be more correct to check localization by application of C- and N-terminal GFP fusions control of native promoter. Secondly, the authors did not used co-localization assay to co-localize GFP-tagged GmKEA5 with strong a marker of the plasma membrane (e.g. PIP protein). Also, the presented picture of GFP distribution did not show a clear localization on the PM. Another concern is that the same work has also attributed the GFP-tagged Glyma09g0213 that encodes a typical endomembrane specific NHX-like (81.2% identity with AtNHX5) transporter to the PM (Chen et al., 2015). Thus, stronger evidence is required to attribute KEA5 localization to the PM in soybean.

In *Triticum aestivum* L., expression profiling of the *TaKEA* family which contains 24 members, implying that they may function in tissue development and response to stress (Sharma et al., 2020).

### CHX Subfamily

The CHX transporters mediate K^+^, Na^+^, H^+^ and, possibly, Cl^-^ transport (Sze et al., 2004; Qu et al., 2020). Most of the CHX members consist of 10–12 transmembrane domains (**Figure 1**). The hydrophobic N-terminal of plant CHX is remarkably similar to cation/proton antiporter-2 KHA1 from yeast (*Saccharomyces cerevisiae*) and *Synechocysti*s NhaS4, indicating potential role of these protein in K+ transport (Sze et al., 2004). Many aspects of CHX topology remains obscure. In general, CHXs consist of around 800 amino acid residues and comprised of 12 transmembrane domains (**Figures 1**, **2**). Several studies have shown that CHXs proteins comprise a Na^+^/H^+^ exchange domain in the N terminus and AANH_like domain (adenine nucleotide alpha hydrolase) close to the C terminus (Padmanaban et al., 2007; Czerny et al., 2016; Jia et al., 2017). The structural models of AtCHX 17 were recently reconstructed in details (Czerny et al., 2016). AtCHX17 and AtNHX1 predicted to share a similar transport domain. However, AtCHX17 exhibit different hydrophilic C-tail of unknown function. Although the truncation of the AtCHX17 C-tail does not affect protein activity, the processes of its sorting and localization in plant cells were inhibited (Chanroj et al., 2013). Structural studies of the AtCHX17 tail domain revealed an architecture similar to the potential protein interaction domain of the bacterial universal stress protein (Czerny et al., 2016). Recently, another conserved domain in the long C-tail of soybean CHXs was found (Jia et al., 2017). Although the function of this domain remains unknown, it was suggested to be involved in a phosphorylation or protein localization in a manner similar to NHX family (Jia et al., 2017). Thus, the question regarding regulation and protein interaction with other partners remains open and needs to be solved in further studies on CHX functions.

**Figure 6:**
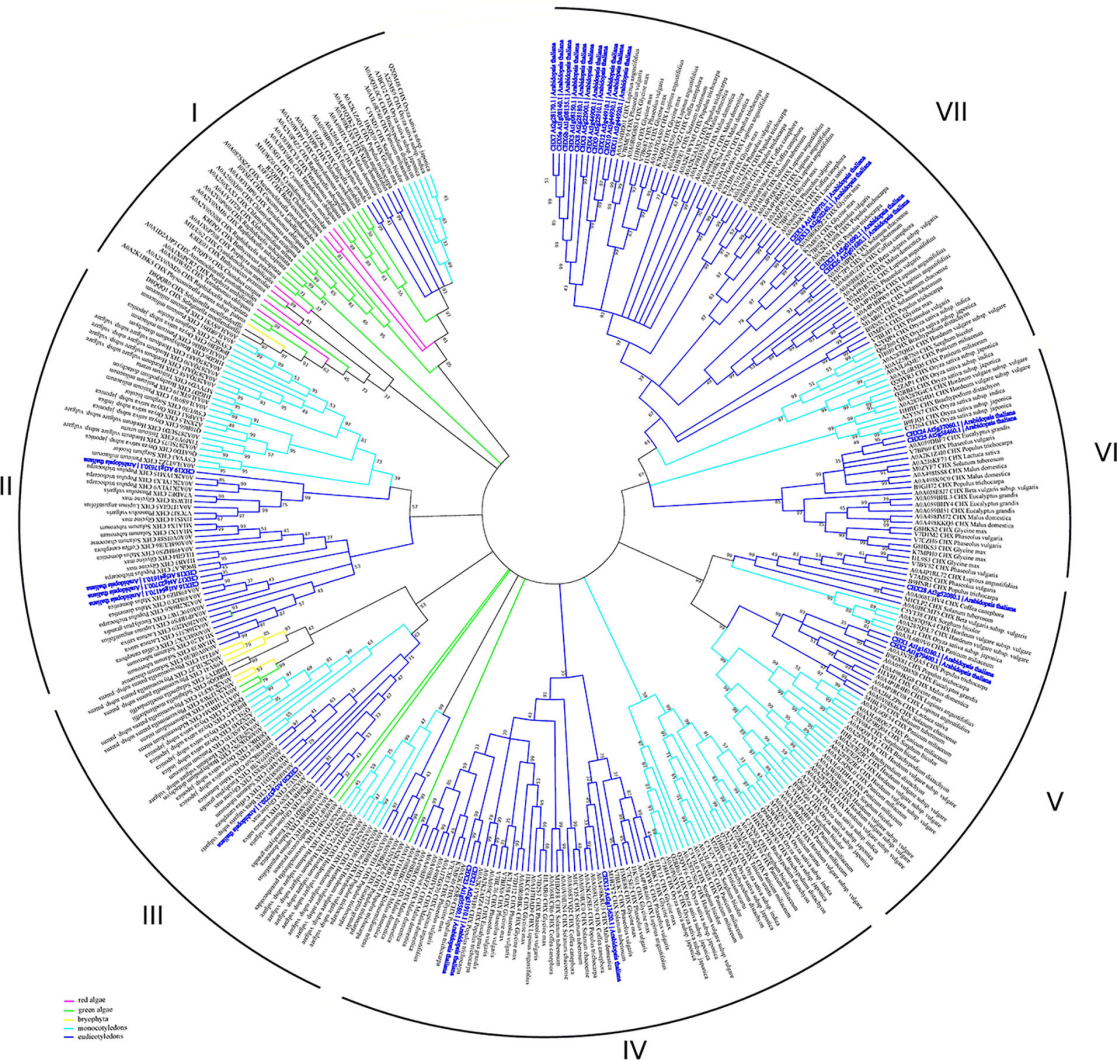
Phylogenetic analysis of CHX proteins. The Maximum Likelihood method and “Dayhoff” model were used; 1,000 bootstrap replicates. The phylogenetic tree was obtained with MEGA X software on the basis of full-length protein sequences. Branches colours are: magenta, red algae; green, green algae; yellow, bryophyta; light blue, monocotyledons; blue, eudicotyledons. *Arabidopsis thaliana* sequences are highlighted in bold blue.

CHX transporters are widely distributed among bacteria, fungi, and plants (Chanroj et al., 2012). However, this type of proteins is not found in animals. In contrast to other members of CPA subfamilies, the flowering plants have a wide range of CHX proteins. Arabidopsis genome encodes 28 members of CHXs, rice has 17, *Glycine soja* has 34, while *Physcomitrella patens* has only four CHX members (Mottaleb et al., 2013; Jia et al., 2017). A large number of CHXs members exhibit pollen specificity and play an important role in the pollen growth and flower development *via* maintaining of K^+^ homeostasis (**Table 2**) (Sze et al., 2004; Chen et al., 2016; Zhou et al., 2016; Jia et al., 2017). Interestingly, 18 out of 28 CHX genes in *Arabidopsis* are pollen specific or pollen enhanced, and only six genes were found to be expressed in vegetative tissues (Sze et al., 2004). Besides pollen development, various members of CHX are involved in K^+^/Na^+^ transport, osmotic adjustment, stomatal opening, and salt tolerance (Sze et al., 2004; Padmanaban et al., 2007; Jia et al., 2018). According to early phylogenetic analyses, the CHX family were divided into five groups, highlighting the largest subclades Group IV, consisting of eight members (AtCHX15-21 and AtCHX23) (Sze et al., 2004). The further phylogenetic analysis of CHX members from 14 different plant species revealed significant diversification of CHX family in flowering plants. Overall, CHX genes are less diverged in monocots than in dicots (Chanroj et al., 2012). The average number of CHX genes in the genome of monocots is 16 while dicots genome encoded at least 26 genes (Chanroj et al., 2012). In the latter group, CHX are classified into 8 different subclades, while in monocots only 5 subclades are present (Chanroj et al., 2012). Our analysis of CHX phylogenetic relation in plant kingdom suggests that the existence of seven different clades (**Figure 6**). In contrast to other CPA families, CHX contains the highest number of proteins (**Figure 6**, **Table S1**). Similar to the NHX family (groups NHX1-4 and NHX5-6), CHX family is formed only by the sodium/hydrogen exchanger domain, without any additional domains or signaling peptides identified (**Figure 1**). In contrast to previous studies, we have significantly extended number of plant species, to include green and red algae into analysis. Therefore, our phylogenetic analysis displaying existence seven clades instead of five suggested before. Apparently, CHX family plays an important physiological role, and was subjected for multiple duplication and specialization events, especially in mono- and dicotyledon species. As a result of this, five (II, IV, V, VI, and VII) of seven defined clades are formed only with mono- and dicotyledon species (**Figure 6**). The CHX members belonging to clade VII represent the most recent and specialized development, being assembled of mostly dicotyledon species. From the other side, Clade I could be described as the most primitive one, combining proteins from red algae (*Cyanidioschyzon merolae, Chondrus crispus*, and *Porphyra umbilicalis*), greed algae (all examined species), Bryophyta (*Physcomitrella patens*), lycophyte (*Selaginella moellendorffii*), and some mono- and dicotyledon species (**Figure 6**, **Table S1**). Transitional clade III includes only some proteins from green algae (*Klebsormidium nitens*, *Bathycoccus prasinos* and *Auxenochlorella protothecoides*) (**Figure 6**). Also, the number of the CHX and lycophyte is much smaller in comparison to the mono- and dicotyledon species, and limited to 1-6 proteins. The CHX members from Arabidopsis are distributed among six different clades (II, III, IV, V, VI, VII) (**Table 2**, **Figure 6**). Thus, our study suggests that primary role of primitive CHX transporters from clade I is likely related to the maintenance of cation homeostasis in the cells. Overall, it appears that the diversification of CHX members in plant kingdom is even more complex than it was assumed before.

**Table 2:** Selected examples of the properties, tissue-specific expression, and physiological functions of CHX transporters in plants.

CHX isoform and plant species	Clade	Tissue expression	Membrane localization	Transport properties	Demonstrated function	References
In *Arabidopsis thaliana*						
AtCHX1 AtCHX2	V	Pollen		K^+^?		Sze et al., 2004; Bock et al., 2006
AtCHX3 AtCHX4 AtCHX5 AtCHX6a AtCHX6b	VII	Pollen		K^+^?		Sze et al., 2004; Bock et al., 2006
AtCHX8 AtCHX9						
AtCHX10	VII	Pollen/Root		K^+^?	Downregulation by salinity.	Maathuis et al., 2003; Sze et al., 2004; Bock et al., 2006
AtCHX11 AtCHX12	VII	Pollen		K^+^?		Sze et al., 2004; Bock et al., 2006
AtCHX13	VII	Pollen, root tip and elongation zone	PM	K^+^	High-affinity K^+^ transporter; K^+^ uptake under potassium deficiency.	Zhao et al., 2008; Zhao et al., 2015
AtCHX14	VII	Pollen	PM	K^+^	Low K^+^ affinity transporter; K^+^ homeostasis and K^+^ recirculation in plants.	Zhao et al., 2008; Zhao et al., 2015; Jia et al., 2018
AtCHX15	IV	Pollen, leaves, root		K^+^?	Downregulation by salinity	Maathuis et al., 2003; Sze et al., 2004,
AtCHX16	II	Root/leaf	ER	K^+^		Chanroj et al., 2011;
AtCHX17	II	Epidermal and cortical cells of mature roots	PVC in plant, PM in plant, Golgi apparatus in plant and yeast	K^+^	pH and K^+^ homeostasis in Golgi cisternae; protein sorting; salt tolerance, embryo development, pollen wall formation, male fertility.	Cellier et al., 2004; Sze et al., 2004; Maresova and Sychrova, 2006; Chanroj et al., 2011; Chanroj et al., 2013; Sun et al., 2015; Czerny et al., 2016; Padmanaban et al., 2017
AtCHX18	II	Root/Leaf	PVC	K^+^	Embryo development; pH and K^+^ homeostasis affect pollen wall formation, male fertility.	Chanroj et al., 2011; Padmanaban et al., 2017; Jia et al., 2018
AtCHX19	II	Root, leaf	PVC	K^+^	Cation/proton exchanger at an early phase of male gametogenesis.	Sze et al., 2004; Chanroj et al., 2011; Padmanaban et al., 2017
AtCHX20	III	Guard cells, root tip/cap	ER	K^+^	Osmoregulation, salt stress responses; light-induced stomatal opening by regulating K^+^ flux and by pH modulation.	Padmanaban et al., 2007; Chanroj et al., 2011; Padmanaban et al., 2017
AtCHX21	IV	Root endodermal cells, leaves, pollen	PM	Na^+^ and K^+^?	Na^+^ transport into the xylem; pollen K^+^ homeostasis; salt stress responses, pollen tube navigation to the ovule.	Hall et al., 2006; Evans et al., 2012; Lu et al., 2011
AtCHX23	IV	Leaves; pollen	ER in pollen tube	K^+^ and Na^+^	Osmotic adjustment and K^+^ homeostasis of pollen, pollen tube navigation to the ovule	Song et al., 2004; Sze et al., 2004; Evans et al., 2012; Lu et al., 2011
AtCHX24 AtCHX25	VI	Pollen		K^+^?		Sze et al., 2004; Bock et al., 2006
AtCHX26 AtCHX27	VII	Pollen		K^+^?		Sze et al., 2004; Bock et al., 2006
AtCHX28	V	Pollen		K^+^?		Sze et al., 2004; Bock et al., 2006
In *Oryza sativa* L.						
OsCHX11	III	Roots		K^+^	Upregulation by salinity; salt tolerance.	Senadheera et al., 2009
OsCHX14	II	Lodicules and the region close by throughout the flowering process	ER	K^+^, Rb^+^, Cs^+^	K^+^ homeostasis during rice flowering.	Chen et al., 2016
In *Glycine max*						
GmCHX1 (GmSALT3)	III		ER	Na^+^, Cl^-^?	Salt tolerance.	Guan et al., 2014; Qi et al., 2014; Qu et al., 2020
GmST1	III	Leaves		Na^+^ and K^+^?	Salt and drought tolerance ABA-dependent.	Ren et al., 2016
In *Glycine* *soja*						
GsCHX19.3	II	Root, leaf, flower	PM	K^+^	K^+^ uptake; salt and carbonate alkaline tolerance	Jia et al., 2017
In *Pyrus bretschneideri*						
PbrCHX16	IV	Pollen	PM	K^+^	Pollen tube growth	Zhou et al., 2016
In *Physcomitrella patens*						
PpCHX1	III		Golgi	K^+^	K^+^ homeostasis	Mottaleb et al., 2013
PpCHX2	III		PM, Vacuole	K^+^	K^+^ homeostasis; transfer of K^+^ from the vacuole to the cytosol or from the cytosol to the external medium.	Mottaleb et al., 2013

Interestingly, some studies reported that CHX functions may partially overlap with the functions of HAK, NHX, and KEA transporters (Pardo et al., 2006; Grabov 2007; Chanroj et al., 2012; Haro et al., 2013; Sze and Chanroj, 2018). Some authors suggested that plant species with a high number of CHX genes have a lower number of HAK genes and *vice versa* (Yang et al., 2009; Chanroj et al., 2012). However, our view is that evolutional multiplication of CHX genes is primarily related to the appearance of the complex pollination process in dicotyledonous flowering plants.

Several studies suggested the participation of CHXs in plant salt stress response, *via* maintenance of K^+^ homeostasis (Jia et al., 2018; Qu et al., 2020). We have to note, that cytosolic K^+^ retention is one of the key traits conferring salinity tissue tolerance in root and leaf tissues (Shabala and Pottosin, 2014; Wu et al., 2018; Rubio et al., 2020). In addition, there is a growing amount of evidences suggesting that cytosolic K^+^ may operate as another second messenger mediating plant adaptive responses to hostile environment, including salinity (Anschütz et al., 2014; Shabala, 2017; Adem et al., 2020). Thus, given K^+^ transport ability of CHX proteins, they could be an important part of machinery regulating cytosolic K^+^ homeostasis and shaping K^+^ “signatures” in response to salt stress (Rubio et al., 2020). The salt tolerance mediated by CHXs could be achieved by several possible strategies: tissue K^+^ distribution in favor of active metabolic tissues under salinity stress; K^+^ deposition and accumulation in cellular endomembrane compartment; generation of membrane proton gradient *via* H^+^ exchange and subsequent recruitment of ion channels and pH cytosolic adjustment during salinity stress. Moreover, some CHXs are might be involved in Na^+^ transport. Therefore, these type of transport properties could be substantial for Na^+^ tissue and cellular removal and vacuolar or endosomal sequestration, Na^+^ redistribution between different type of tissues in accordance to plant metabolic and physiological needs (Isayenkov and Maathuis, 2019).

Although the specific details may be still lacking, the involvement of AtCHX17, AtCHX20 and AtCHX21 in plant salt stress response was demonstrated (**Table 2**). In addition, all known and characterised CHX members (GmCHX1, GmST1, and GsCHX19.3) from soybean (*Glycine max and G. soja*) are believed to be involved in plant salt tolerance responses (Guan et al., 2014; Qi et al., 2014; Ren et al., 2016; Jia et al., 2017; Qu et al., 2020) (**Table 2**). For example, pre-vacuolar compartments (PVC) and plasma membrane (PM) localized AtCHX17 might be one of key player of maintaining ionic homeostasis under salinity stress and K^+^ starvation (Chanroj et al., 2011; Chanroj et al., 2013). Moreover, the *AtCHX17* expression was highly induced by acidic pH, ABA, Ca^2+^ deprivation, osmotic stress and cold, suggesting the importance of this transporter in plant adaptation to a wide range of abiotic stresses (Maathuis et al., 2003; Cellier et al., 2004; Pardo et al., 2006). Interestingly, AtCHX17 and AtNHX1 are predicted to share similar protein architecture and transport core; however, they have differences in their pH dependence and thus could play different roles in cation homeostasis and membrane trafficking (Sze and Chanroj, 2018).

Some members of CHX family displayed ability for Na^+^ and even possible Cl^-^ transport, thus potentially contributing to plant responses to salinity (Hall et al., 2006; Evans et al., 2011; Qi et al., 2014; Jia et al., 2017; Jia et al., 2018; Qu et al., 2020) (**Table 2**). This specifically includes AtCHX21 and AtCHX24 transporters (Hall et al., 2006) (**Table 2**, **Figure 3**). The mutant analysis of CHX21 revealed that seedlings carrying a knockout of *Atchx21* grow more slowly in the presence of 50 mM and 100 mM NaCl (Hall et al., 2006). The early studies suggested that CHX21 is likely to be K^+^/Na^+^/H^+^ antiporter involved in xylem Na^+^ (K^+^) loading and, consequently, Na^+^ accumulation in the leaf (Hall et al., 2006). The follow-up studies suggested that this transporter is involved in K*^+^* transport and homeostasis (Evans et al., 2011; Lu et al., 2011). However, the direct experimental evidences of the transport properties of CHS21 are lacking, with all complementation assay work conducted on CHX23 (Hall et al., 2006; Lu et al., 2011) but not CHX21.

Also, early study of AtCHX23 functionality suggested its role in plant response to salinity (Song et al., 2004). The detailed analysis of the loss-of-function mutant and the cellular localization suggested that AtCHX23 is Na^+^(K^+^)/H^+^ exchanger that is involved in the adjustment of pH level in the chloroplast stroma. It was also reported that AtCHX23 is localized in the plastid envelope and tissue expression pattern comprised, root, leaves stem and flowers (Song et al., 2004). More recently, two studies have shown that AtCHX21 and AtCHX23 could be essential components of the pollen tube development. The *chx21* or *chx23* mutants did not show any clear phenotype. In contrast to this, the double *chx21 chx23* mutant exhibited impaired male fertility (Lu et al., 2011) (**Table 2**). Under the control of its native promoter, the GFP-tagged AtCHX23 was localized to the ER of pollen tubes, but not plastids and deemed to be a pollen specific K^+^ transporter. It very likely that AtCHX23 involved in K^+^ uptake and growth in a pH-dependent manner (Lu et al., 2011). Perhaps, the potential problems with such differences in reported functions of AtCHX23 is that *chx23* mutant alone has no phenotype (Lu et al., 2011). Taking into the account that RT-PCR and RNA-seq analysis shows high level of AtCHX23 in pollen, it is questionable whether the early study conducted by Song et al., 2004 has been used the same gene.

The biological functions of CHXs are wide and could comprise stomata movement, embryo development, male fertility, and other pivotal physiological processes. For example, it was found that AtCHX20 is a key component of light-induced stomatal opening (Padmanaban et al., 2007). The CHXs are localized in the different types of cellular membranes (**Table 2**, **Figure 3**). For example, AtCHX18 and AtCHX19 are found in PVC, whereas AtCHX16 and AtCHX20 possess endoplasmic reticulum (ER) specificity (Chanroj et al., 2011). In addition, detailed analysis of the triple c*hx17chx18chx19* mutant revealed a crucial role of CHX17-19-encoded K^+^/H^+^ antiporters in pH and K^+^ homeostasis and wall formation in pollen and male fertility (Padmanaban et al., 2017) demonstrating that AtCHX17 and AtCHX18 proteins may facilitate embryo development. Interestingly, AtCHX17 is involved in regulation of membrane trafficking and cargo delivery and protein sorting (Padmanaban et al., 2017); the processes pivotal for the male fertility, sperm function, and embryo development (**Table 2**).

Some members of CHXs are targeted to plasma membrane, however exact physiological role of transporters is still remains largely uncharacterised. For example, AtCHX13, AtCHX14, and AtCHX21 exhibited plasma membrane localization, while PpCHX1 from *Physcomitrella patens* is Golgi specific, and PpCHX2 is localized at the tonoplast and plasma membranes (**Table 2**, **Figure 3**).

An interesting funding have been recently reported by Qu et al. (2020) who have studied functionality of a *Glycine max* GmCHX1 (GmSALT3) gene that is closely related to *A. thaliana* AtCHX20 (**Table 2**). The authors provided some evidence suggesting that phloem translocation of Cl^–^ could be mediated by GmCHX1 thus implying its role in long-distant Cl^-^ transport and adaptation to salinity. This suggestion, however, needs to be validated in future experiments.

Taking together, the CHX transporters were significantly diversified during evolution of plants. The process of diversification is reached its highest point in the flowering plants. The large part of CHXs of higher plant is exhibit specific functioning in plant reproductive organs. However, this group of membrane transporters is also important for plant tolerance to abiotic stress and, specifically, soil salinity.

## Conclusions

CPA gene superfamily encompasses a wide range of plant membrane transporter involved in monovalent ion transport across cellular membranes. It is believed that members of CPA1 proteins are critical in mediating plant salt tolerance and have been evolved to maintain cellular cation and pH homeostasis. NhaPs are involved in the regulation of Na^+^ or Li^+^ exclusion at the plasma membrane and were evolved later (as NHX exchangers) for sequestration of Na^+^ and K^+^ in vacuoles and/or endomembrane compartments. Phylogenetic analysis of this group of transport protein revealed that their development and specialization occurred along with land colonisation by plants, after acquiring central vacuoles and efficient use of this cellular compartment for physiological adjustments to constantly changing environment. SOS1 homologues, in their modern form, were formed in algae, while other families (KEA, CHX, and NHX) are much elder and originate from bacteria. Interestingly, the SOS related signaling pathways are considered to regulate activities of SOS1 and (evolutionary more recently) NHX1-4 antiporters, suggesting relative novelty of this signaling mechanism to adapt to salinity, drought and other type of stresses. Despite a wide recognition of their functional roles, regulation of SOS1 and NHX transporters needs to be studied in more depth. In contrast to CPA1, the CPA2 family is present in bacteria, fungi, and plants, whereas is *de facto* absent in mammals (with only one mammalian transporter reported). The development and diversification of CPA2 transporters is especially pronounced in higher plants. For example, an evolutionary appearance of non-chloroplastic KEA 4, 5, and 6 transporters occur only in mono- and dicotyledon species. Moreover, a diversification burst of CHX transporters is closely related to the development of reproductive organs of flowering plants. Thus, higher plants are excellent platform for efficient recruitment, development and further cellular and tissue specialization of CPA transporters. Although the functions of CPA2 family members (KEA and CHX) are studied much less and attributed largely to the reproductive developmental processes, many members of theses transport proteins might potentially play an important role in salt and drought tolerance. This specific topic warrants a stronger focus of the future studies.

The range of novel and modern techniques to study functional features of transport proteins is continuously extending. The application of high-throughput sequencing facilitated identification of CPAs within wider range of organisms, including red and green algae. The further implementation of analytical tools such as synchrotron‐based X‐ray fluorescence (SXRF) imaging techniques will help to get insight into the CPA functions in ion homeostasis, abiotic stress tolerance, and cellular compartmentation in more details (Punshon et al., 2013; Pittman and Hirschi, 2016). Moreover, application of non-invasive NMR-imaging and NMR-spectroscopy may provide a great opportunity to visualise internal tissue structure, distribution of water and ions within the plant tissues in real time (Borisjuk et al., 2012). Electrophysiological microelectrode ion flux-measuring techniques may allow functional studies of transport properties and regulation of specific transporters at the single-cell level, thus providing insights into the tissue-specificity of their operation (Pedersen et al., 2020). Extensive application of plant mutant lines together with widely spreading genome editing techniques are also highly promising to study and characterize CPAs in future. The tools of synthetic biology for the modification of the existing membrane transporters or engineering of the completely novel proteins, with the desired properties, will significantly intensify the process of novel discoveries in plant mineral transport. Overall, the detailed knowledge of evolution, operation, and regulation of CPA transporters may be instrumental in developing new strategies and approaches to improve salt and drought tolerance as well as boost general crop fitness and productivity.

## Author Contributions

All authors contributed to the article and approved the submitted version. SI contributed to Introductory and CHX transporters section, general discussion, and figures and tables. He was also responsible for the general editing of the manuscript. SD has conducted phylogenetic analysis for all CPA transporters, was responsible for preparation of figures, and written a section dedicated to NhaP subfamily. TP was responsible for writing the section dealing with KEA transporters and contributed to **Table 2**. SS has conducted general manuscript editing and written the section dealing with NHX/NHE transporters.

## Funding

This work was supported by China National Natural Science Foundation [Projects 31961143001 and 31870249] and Australian Department of Industry, Innovation and Science [project AISRF48490] grants to SS.

## Conflict of Interest

The authors declare that the research was conducted in the absence of any commercial or financial relationships that could be construed as a potential conflict of interest.

## Supplementary Material

Supplementary Table 1 List of plant species used for phylogenetic analysis.
